# Microsatellite alteration in head and neck squamous cell carcinoma patients from a betel quid-prevalent region

**DOI:** 10.1038/srep22614

**Published:** 2016-03-24

**Authors:** Jin-Ching Lin, Chen-Chi Wang, Rong-San Jiang, Wen-Yi Wang, Shih-An Liu

**Affiliations:** 1 Department of Radiation Oncology, Taichung Veterans General Hospital, Taichung, 40705, Taiwan; 2 Department of Otolaryngology, Taichung Veterans General Hospital, Taichung, 40705, Taiwan; 3 Faculty of Medicine, School of Medicine, National Yang-Ming University, Taipei, 11221, Taiwan; 4 Department of Nursing, HungKuang University, Taichung, 43302, Taiwan

## Abstract

We investigated the frequency of microsatellite alteration and their impact on survival in head and neck squamous cell carcinoma patients from an endemic betel quid chewing area. We collected 116 head and neck squamous cell carcinoma specimens along with corresponding surgical margins which were confirmed by pathological examination. Ten oligonucleotide markers were chosen for the assessment of microsatellite alteration. The specimens were amplified by polymerase chain reaction followed by automatic fragment analysis. There were 44 specimens (37.9%) with microsatellite instability (MSI) in at least one marker while more than half of the specimens (n = 68, 58.6%) had loss of heterozygosity (LOH) in at least one marker. Though MSI/LOH was not correlated with the survival of head and neck squamous cell carcinoma patients, presence of MSI in the tumor-free surgical margins was associated with local recurrence (odds ratio: 15.14; 95% confidence interval: 6.451 ~ 35.53; *P* < 0.001). Genomic assessment of surgical margin can help surgeons to identify head and neck squamous cell carcinoma patients who are at risk of developing local recurrence in a betel quid-prevalent region.

Head and neck cancer is one of the most common cancers with an estimated annual incidence of nearly 600,000 new cases and about 325,000 mortalities worldwide^1^. Furthermore, the incidence rates of head and neck cancer have risen in many countries over the past decade^2^. Although better combinations of loco-regional plus systemic therapeutic modalities have improved the quality of life after treatment, the 5-year survival rate has not changed much in recent years^3^,^4^.

Two types of microsatellite alterations have been reported to correlate with various types of cancer; microsatellite instability (MSI) and loss of heterozygosity (LOH)^5^. Microsatellites are an extension of DNA in which 1–6 base pairs are tandemly repeated 5–100 times and are vulnerable to inappropriate replication during DNA duplication^6^. MSI results from the failure to correct the aforementioned inaccurate repetition producing insertion or deletion of repeated DNA microsatellite sequences^5^,^7^. The prognostic and predictive value of MSI in colorectal cancer has been well documented in the literature^8^. In addition, MSI was reported to be associated with a higher risk of multiple malignancies in head and neck cancer patients^9^. The reported incidence of MSI in head and neck squamous cell carcinoma varies from 3 to 88%^6^,^7^,^9^. De Schutter *et al*. even found only one out of 80 tumors could be considered positive for MSI in advanced head and neck cancer patients^10^. Furthermore, MSI in the surgical margin was found to increase the likelihood of local recurrence in head and neck squamous cell carcinoma patients^7^. However, the predictive value of MSI is debatable and further study is warranted to clarify the relationship between MSI and survival of head and neck cancer patients^6^.

LOH is recognized as the loss of a chromosome loci, microsatellite allele, or single-nucleotide polymorphisms in a tumor specimen when compared with normal tissue^6^. LOH can inactivate tumor suppressor genes and lead to uncontrolled cell growth if it happens in a nearby location^11^. LOH was reported to be related to lymph node metastasis in hypopharyngeal cancer patients^12^. Murali *et al*. found LOH at D9S162 was a poor prognosticator of recurrence-free survival in oral cancer patients^13^. In addition, LOH and genomic instability were also used as a marker of clonality in differentiating true recurrence from second primary tumor in head and neck cancer^14^. MSI and LOH on chromosome 9 have been reported as an early event in oral cancers. The genetic alterations were primarily in the chromosomal region 9p21-23. D9S157, D9S161, and D9S1748 were found to have a higher incidence of microsatellite alteration in the abovementioned region^15^. Arai *et al*. in their study on oral cancers showed that LOH at 3p could be identified in early-stage lesions and the incidence of LOH increased in later clinical stages. Ten informative markers including THRB and D3S1079 were used to detect LOH^16^. Another study which investigated microsatellite alteration on chromosome 3p showed that the highest frequency of LOH was found with markers D3S1234 and D3S1300^17^. However, few studies from betel quid-prevalent regions on MSI and LOH in head and neck squamous cell carcinoma have been reported in the literature. Therefore, we investigated microsatellite alteration and its impact on survival of head and neck squamous cell carcinoma patients in an endemic betel quid chewing area.

## Results

There were 149 potential participants scheduled to undergo surgical resection for head and neck cancer during the study period. Among them, 5 patients (3.4%) refused surgery. In addition, 4 patients (2.7%) declined to participate in the study and 2 patients (1.3%) had a histological type other than squamous cell carcinoma. During the postoperative pathological assessment, 13 patients (8.7%) who had inadequate surgical margins (less than 5 mm) and 9 patients (6.0%) with various degrees of dysplasia in at least one of the mucosa margins were also excluded from the final analysis. Comprehensive information was acquired from 116 patients. The average age of the patients at diagnosis was 52.7 +/- 11.4 years and males accounted for 94.0% (n = 109) of all patients. The majority of patients had a primary site inside the oral cavity (n = 104, 89.7%), followed by the oropharynx (n = 7, 6.0%). With respect to personal habits, 92 patients (79.3%) were smokers, 84 patients (72.4%) consumed alcohol socially or heavily, and 83 patients (71.6%) habitually chewed betel quid. Thirty-two patients (27.6%) had stage I diseases, whereas 21 (18.1%), 12 (10.3%), and 51 (44.0%) patients had stage II, III, and IV diseases, respectively. Fifteen patients (12.9%) died and 26 patients (22.4%) developed local recurrence during the study period. All 15 patients died because of extensive local recurrence or complications during salvage treatment. In addition, 3 patients (2.6%) had neck lymph node recurrence and no patient developed distant metastasis. The average follow-up period was 21.9 months ( + 10.6 months). All participants except one had no traceable family history. One participant’s father had also been diagnosed with oral squamous cell carcinoma.

In total, 37.9% (44 out of 116) of the cancerous specimens had MSI in at least one marker. The most frequently positive marker for MSI was D21S236 (n = 14, 31.8%), followed by IFNA.PCR2 (n = 9, 20.5%), and THRB (n = 8, 18.2%). In terms of LOH, more than half of the specimens (n = 68, 58.6%) had LOH in at least one marker. The most frequently positive marker for LOH was IFNA.PCR2 (n = 35, 51.5%), followed by D9S1748 (n = 23, 33.8%), and D3S1300 (n = 19, 27.9%).

**Figure 1:**
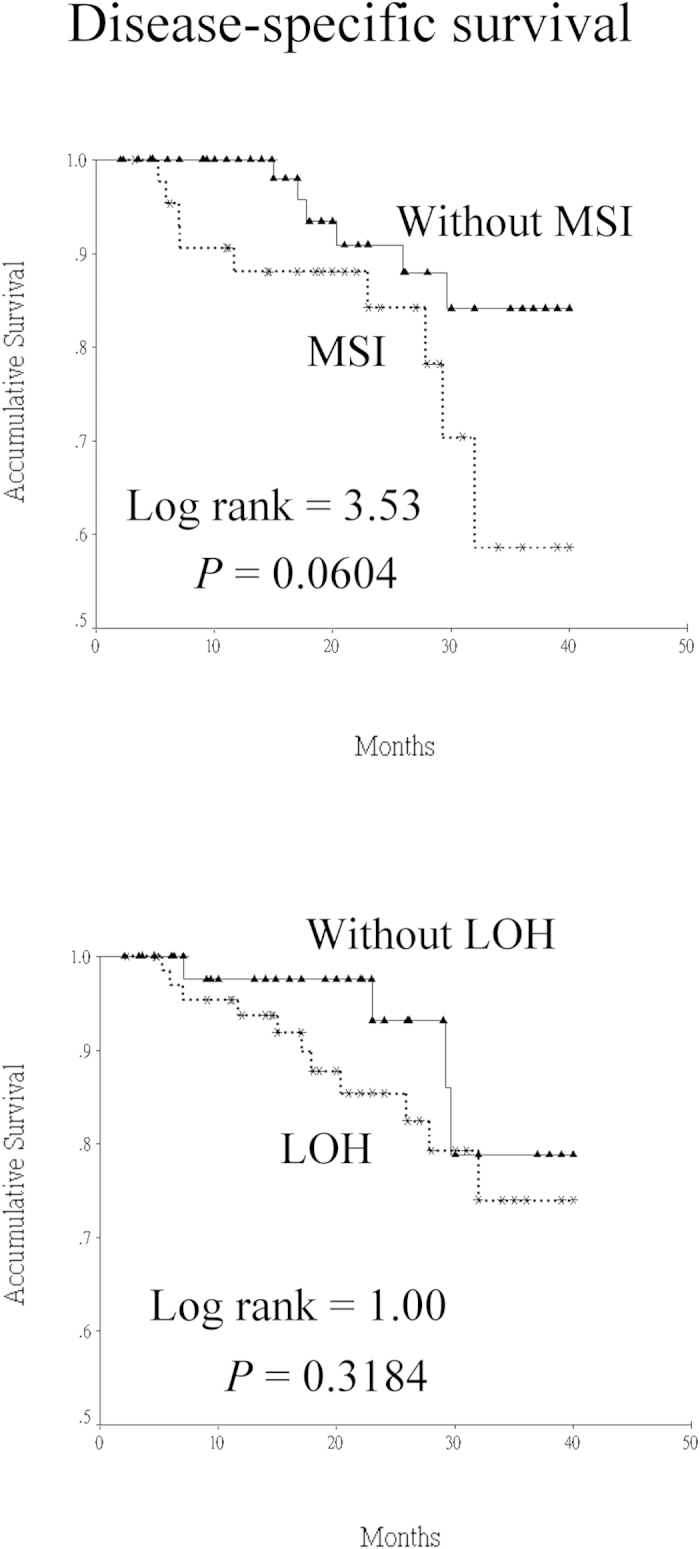
Disease-specific survival curves of head and neck squamous cell carcinoma patients based on the status of microsatellite alteration. (MSI: microsatellite instability; LOH: loss of heterozygosity).

All patients were then divided into 2 groups based on the status of microsatellite alterations. Comparisons of variables between the 2 groups are presented in Table 1 and 2. Although participants with MSI tended to have a higher rate of local recurrence and mortality when compared with those without, the differences did not reach statistical significance. Nevertheless, no significant differences were noted between the MSI and non-MSI groups in age, gender, lifestyle habits, histological characteristics, peri-neural invasion, angiolymphatic invasion, pathological stage, and postoperative radiotherapy. Furthermore, there were also no statistical differences in all variables between patients with LOH and those without. An analysis of the habitual smokers revealed that the quantitative data were similar between patients with MSI/LOH and those without (MSI vs. non-MSI: 31.0 +/- 21.7 vs. 28.8 +/- 15.8 pack-years, *P* = 0.572; LOH vs. non-LOH: 28.6 +/- 14.8 vs. 31.1 +/- 22.2 pack-years, *P* = 0.510). In terms of habitual betel quid chewers, a higher average consumption amount was noted in patients with MSI/LOH when compared with that of those without (MSI vs. non-MSI: 538 +/- 420 vs. 309 +/- 302 quid-years, *P* = 0.009; LOH vs. non-LOH: 435 +/- 415 vs. 357 +/- 294 quid-years, *P* = 0.349). Based on the survival analysis using the Kaplan-Meier method, although a higher 3-year disease-specific survival rate was noted in patients without MSI when compared with that of those with MSI, no significant statistical difference was found. (84.1% vs. 56.7%, *P* = 0.0604). There was also no significant difference in 3-year disease-specific survival between patients with LOH and those without (73.9% vs. 78.8%, *P* = 0.3184) (Fig. 1). In the multivariate analysis, pathological stage was the only independent factor associated with disease-specific survival (Table 3).

A total of 506 specimens were collected from the margins of surgical defect. The abovementioned 506 surgical margins came from 116 patients and 436 of them were mucosa margins whereas 70 of them were deep margins. Our analysis of the preliminary results revealed no microsatellite alteration in the surgical margins from the tumor specimens which had no detectable microsatellite alteration. Temam *et al*. in their study also excluded margins from tumors without microsatellite instability in the final analysis^7^. Therefore, we decided to stop further analysis of surgical margins from patients without microsatellite alteration in tumor specimens after a thorough discussion among the authors. Finally, 351 surgical margins from 79 informative patients were included in the logistical regression model. Ideally, there should be 4 mucosa margins and one deep margin per patient. However, when we excised buccal tumor along with cheek skin, there was no deep margin. In addition, when we excised buccal tumor including mouth angle, there was no anterior mucosa margin. Therefore, not all 79 informative patients had 5 margins. Among them, 5 complete surgical margins were obtained from 35 patients whereas only 4 surgical margins were obtained from 44 patients (average 4.44 margins per patient). Among them, 40 margins (11.4%) were found to have MSI whereas 74 margins (21.1%) had LOH in at least one marker. Among the 26 patients who developed local recurrence, 35 corresponding margins were identified as being adjacent to the location of recurrence. The percentage of MSI in the abovementioned margins was higher when compared with that of those without local recurrence (18 of 35, 51.4% vs. 22 of 316, 7.0%, *P* < 0.001). Although the proportion of LOH in the aforementioned margins was also higher when compared with that of the margins without local recurrence, no statistical significance could be found (11 out of 35, 31.4% vs. 63 out of 316, 19.9%, *P* = 0.173). The logistic regression model revealed that late-stage [odds ratio (OR): 2.520; 95% confidence interval (CI): 1.026 ~ 6.186; *P* = 0.044] and presence of MSI in the surgical margin (OR: 15.14; 95% CI: 6.451 ~ 35.53; *P* < 0.001) were independent factors associated with local recurrence. Detailed data are shown in Table 4.

## Discussion

We found 37.9% of head and neck squamous cell carcinoma specimens had MSI, whereas 58.6% of them had LOH in at least one marker. The frequency of MSI reported in the literature ranged from 1.25 to 88% while the frequency of LOH varied from 26 to 100%^6^,^7^,^9^,^10^,^11^,^12^,^13^,^15^,^16^,^17^. The diverse frequency in microsatellite alteration might be explained by the different microsatellite markers selected. In addition, the different composition of the studied population and the different kinds of predisposing factors in disparate regions may also account for the inconsistent findings. Oral carcinogenesis might be initiated by the accumulation of genetic modification that progresses over a period of several years, which encompasses inactivation of the tumor suppressor gene^6^.

Although microsatellite alteration was not related to the survival of head and neck cancer patients, the existence of MSI in the tumor-free surgical margin was associated with a higher rate of local recurrence. A previous study had comparable results despite slight differences in the studied populations and methodologies^7^. MSI is thought to involve dysfunction of the mismatch repair system (MMR), which corrects mistakes during DNA duplication^5^. MMR deficiency may cause carcinogenesis because of accumulative mutations in pivotal genes. Progressive accumulation of MSI was anticipated during tumor growth as invasive carcinomas exhibited more MSI than premalignant lesions^6^. Premalignant lesions in which MSI was present were prone to progress to head and neck cancer^18^. This may explain the 15-fold increased risk of local recurrence in head and neck cancer patients with MSI in surgical margins when compared to those without in the present study.

Brennan *et al*. first described the concept of molecular assessment of surgical margins in head and neck cancer patients using the P53 gene mutation as a molecular marker in 1995^19^. Eukaryotic protein synthesis initiation factor (eIF4E) was also used as a biomarker to assess surgical margins^20^. A major weakness of the aforementioned study is that overexpression of eIF4E is not specific to malignant cells^21^. A combination of methylated genes of endothelin receptor type B and homeobox protein A9 in deep surgical margin was linked to poor locoregional recurrence-free survival in head and neck cancer patients^22^. Nevertheless, none of the candidate markers alone was associated with locoregional recurrence in the abovementioned study. Furthermore, the specificity of DNA methylation alteration for specific tumors is equivocal^20^.

Colorectal cancer patients with MSI have a significantly better survival rate when compared with that of those without^8^. Conversely, the clinical significance and incidence of MSI are variable in tumors other than colorectal cancer^5^. The prognostic effect of MSI in head and neck cancer is still controversial, which is possibly due to the lack of statistical power in previous studies with a generally low incidence of MSI in head and neck cancer^6^. However, it is interesting to note that a higher average amount of betel quid consumption was found in patients with MSI when compared with that of those without in the current study. Zienolddiny *et al*. also found patients with genomic alteration in tumor DNA had a two-fold higher consumption of betel quid than patients without. Therefore, in addition to causing MMR system deficiency, betel quid is thought to induce genomic instability which eventually initiates carcinogenesis^23^. A previous study indicated LOH at D9S162 was a poor prognosticator of recurrence-free survival in oral cancer patients^13^. However, our study failed to demonstrate an association between LOH and prognosis of head and neck cancer. Yalniz *et al*. found that the use of dissimilar microsatellite markers may lead to different frequencies even in the same type of cancer due to unstable sensitivity of individual markers to detect microsatellite alteration^24^. Another explanation of the differences observed in our study compared with various other studies is the different methodologies used. The abovementioned study resolved PCR amplified product in polyacrylamide gels and visualized them by silver staining, while our study used modern automatic fragment analysis procedures, which were reported to offer a more accurate and quantitative assessment when compared to gel electrophoresis^6^. Additionally, the cutoff value of tumor imbalance factor (or LOH ratio) in the current study was less than 0.67 or more than 1.5, while other studies used dissimilar cutoffs such as <0.5 or >2^6^. Tabor *et al*. reported that the genetically altered margin (LOH) in head and neck cancer patients might explain the high risk of local recurrence and second malignancy^25^. However, dysplasia was also found in the margin with LOH in the abovementioned study. In contrast, our study excluded those margins with all grades of dysplasia in order to reduce this confounding effect. Nonetheless, our study failed to demonstrate a relationship between LOH in the surgical margin and local recurrence.

There were some limitations in our study. First, the external validity of the findings is limited as it was conducted at a single institute. Second, the statistical power was probably low due to the relatively small sample size. Third, we only collected MSI/LOH rather than tumor suppressor gene (such as P53) or gene methylation status in specimens. Furthermore, the follow-up period was too short to determine the survival benefit of MSI. Lastly, although the therapeutic guidelines are standardized in our institute, inevitably there were individual differences among patients.

In conclusion, MSI and LOH in the microsatellite markers selected herein were not correlated with survival in head and neck squamous cell carcinoma patients from an endemic betel quid chewing area. The presence of MSI in the surgical margin was associated with local recurrence in head and neck squamous cell carcinoma patients. Further study with a longer follow-up period and a larger population is warranted in order to clarify the relationship between microsatellite alterations and prognosis in head and neck squamous cell carcinoma patients.

## Materials and Methods

This prospective study was approved by the Institutional Review Board of Taichung Veterans General Hospital. Head and neck cancer patients who were scheduled to undergo surgical resection from April 2012 to July 2015 were eligible for recruitment in the present study. All participants were well informed and written consent was acquired before enrollment. Patients who refused surgery, had a histological type other than squamous cell carcinoma, had inadequate chart records, or declined to participate in the study were excluded. Pathological staging was done in accordance with the guidelines of the American Joint Committee on Cancer (7th edition, 2009). The definition of local recurrence was pathological evidence of squamous cell carcinoma located adjacent to the index tumor after comprehensive treatment^14^. Patients who smoked cigarettes or chewed betel quid on a regular basis were documented along with quantitative data. One pack-year was defined as 20 cigarettes (1 pack) per day for 1 year, whereas 1 quid-year was equivalent to chewing one betel quid per day for 1 year. Those who smoked cigarettes, drank, or chewed betel quid only on special occasions such as wedding banquets, family reunions, or birthday parties were not considered habitual users. As different kinds of alcoholic beverages were consumed, patients were categorized according to frequency of use, as follows: non-users, social users, and heavy users. Therapeutic protocols for all participants were conducted in accordance with the consensus guidelines of the head and neck cancer team of our hospital.

### Human head and neck squamous cell carcinoma tissues, surgical margins, peripheral blood, and DNA extraction

Histologically confirmed head and neck cancer specimens were obtained from participants. After en-bloc resection of tumor, we collected surgical margins from the edges of surgical defect including mucosa and deep tissues. The specimens were all stored in liquid nitrogen immediately. Peripheral blood (10 ml) was drawn before operation and was placed in an EDTA-treated tube. Then the sample was centrifuged at 1000 × g for 15 minutes, and the plasma was transferred to 1.5-ml microtubes. The peripheral blood mononuclear cell layer was transferred into a clean 50 ml centrifuge tube, washed twice with a balanced salt solution, and centrifuged at 150 × g for 10 minutes. The samples were stored at −30 °C until use. Total DNA was extracted using the QIAamp DNA Mini kit (QIAGEN) according to the manufacturer’s instructions. The final DNA was dissolved in doubly distilled water and frozen at −30 °C until further processing. After comprehensive pathological assessment, patients with inadequate surgical margins (less than 5 mm, both mucosa and deep margins) or with various degrees of dysplasia in at least one of the mucosa margins were excluded from the final analysis.

### Analysis of MSI/LOH

**Figure 2:**
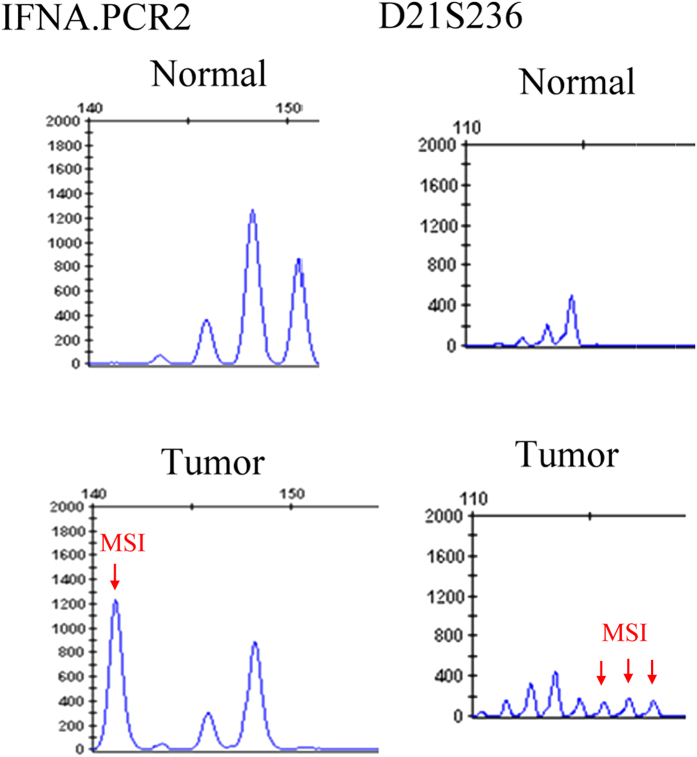
Representative sample of microsatellite instability (MSI) in selected microsatellite markers.

Five binucleotide microsatellites (D9S1748, D3S1079, THRB, D3S1234, D3S1300) were selected based on a literature review^15^,^16^,^17^. Three additional binucleotide microsatellites (IFNA.PCR2, D2S206, D21S236) and 2 tetranucleotide microsatellites (D21S1433, D21S11) were also selected according to the results of previous research conducted in our laboratory (Supplementary Table 1). Multiplex PCR reactions were performed with fluorescent-labeled forward primers and the amplified PCR products were analyzed by capillary array electrophoresis and GeneScan software (Applied Biosystems Inc., Foster City, USA). All the PCR products were purified and sequenced with an ABI Big Dye Terminator (version 3.1) cycle sequencing ready reaction kit and an ABI PRISM 3100 sequencer (Applied Biosystems Inc., Foster City, CA). MSI was defined as the presence of novel fragment sizes in DNA from tumor which was absent in the DNA of leukocytes from peripheral blood (Fig. 2). In addition, the ratio of both microsatellite alleles (allele 2/allele 1) in the peripheral blood leukocyte DNA was divided by the corresponding ratio found in tumor DNA, thus providing the tumor imbalance factor^17^. A value of the aforementioned factor less than 0.67 or more than 1.5 was classified as LOH. At least two independent experiments were employed to confirm the results in each event presenting with MSI/LOH.

### Statistical Analysis

Demographic data were presented as descriptive statistics. In addition, comparisons of continuous variables between subgroups were analyzed by Student’s *t* test, whereas nominal or ordinal variables were analyzed using the Chi-square test or Fisher’s exact test. Survival analysis was analyzed by the Kaplan-Meier method and the differences among subgroups were examined by the log-rank test. Furthermore, relevant factors influencing the survival period were examined by the Cox proportional hazard model. Finally, a backward stepwise logistic regression model was applied to identify pertinent factors associated with local recurrence. All analyses were computed by SPSS for Windows, version 12.1 (SPSS, Chicago, IL) and a *p* < 0.05 was considered statistically significant.

## Additional Information

**How to cite this article**: Lin, J.-C. *et al*. Microsatellite alteration in head and neck squamous cell carcinoma patients from a betel quid-prevalent region. *Sci. Rep*. **6**, 22614; doi: 10.1038/srep22614 (2016).

## Supplementary Material

Supplementary Information

## Figures and Tables

**Table 1:** Descriptive and bivariate analysis of head and neck squamous cell carcinoma patients with or without microsatellite instability (MSI).

Variables	Total no. of patients (% in column)	No. of patients (%)		*P* value
		MSI (n = 44)	Without MSI (n = 72)	
Age (yr)		50.5 +/- 10.1	54.0 +/- 12.0	0.108
BMI (Kg/M^2^)		24.2 +/- 3.9	25.2 +/- 4.5	0.206
F/U duration (month)		22.0 +/- 9.6	22.0 +/- 11.3	0.988
Gender (Female/Male)	7/109	3/41	4/68	0.999†
Smoking				0.851
Yes	92(79.3%)	34(37.0%)	58(63.0%)	
No	24(20.7%)	10(41.7%)	14(58.3%)	
Alcohol				0.208
Heavy	37(31.9%)	16(43.2%)	21(56.8%)	
Social	47(40.5%)	20(42.6%)	27(57.4%)	
No	32(27.6%)	8(25.0%)	24(75.0%)	
Betel quid				0.392
Yes	83(71.6%)	34(41.0%)	49(59.0%)	
No	33(28.4%)	10(30.3%)	23(69.7%)	
Primary tumor sites				0.999†
Oral cavity	104(89.7%)	40(38.5%)	64(61.5%)	
Others	12(10.3%)	4(33.3%)	8(66.7%)	
Histological features				0.736
Well differentiated	8(6.9%)	2(25.0%)	6(75.0%)	
Moderately differentiated	82(70.7%)	32(39.0%)	50(61.0%)	
Poorly or undifferentiated	26(22.4%)	10(38.5%)	16(61.5%)	
Perineural invasion				0.452
Yes	26(22.4%)	12(46.2%)	14(53.8%)	
No	90(77.6%)	32(35.6%)	58(64.4%)	
Angiolymphatic invasion				0.532
Yes	26(22.4%)	8(30.8%)	18(69.2%)	
No	90(77.6%)	36(40.0%)	54(60.0%)	
Extracapsular invasion				0.202†
Yes	11(9.5%)	2(18.2%)	9(81.8%)	
No	105(90.5%)	42(40.0%)	63(60.0%)	
Pathological stage				0.853
Stage I-II	54(46.6%)	20(37.0%)	34(63.0%)	
Stage III-IV	62(53.4%)	24(38.7%)	38(61.3%)	
Postoperative radiotherapy				0.968
Yes	53(45.7%)	20(37.7%)	33(62.3%)	
No	63(54.3%)	24(38.1%)	39(61.9%)	
Local recurrence				0.058
Yes	26(22.4%)	14(53.8%)	12(46.2%)	
No	90(77.6%)	30(33.3%)	60(66.7%)	
Survival status				0.059
Alive	101(87.1%)	35(34.7%)	66(65.3%)	
Death	15(12.9%)	9(60.0%)	6(40.0%)	

^†^Fisher’s exact test.

**Table 2:** Descriptive and bivariate analysis of head and neck squamous cell carcinoma patients with or without loss of heterozygosity (LOH).

Variables	Total no. of patients (% in column)	No. of patients (%)		*P* value
		LOH (n = 68)	Without LOH (n = 48)	
Age (yr)		51.4 +/- 11.0	54.4 +/- 11.9	0.162
BMI (Kg/M^2^)		25.3 +/- 4.4	24.1 +/- 4.0	0.138
F/U duration (month)		22.6 +/- 10.3	21.1 +/- 11.1	0.455
Gender (Female/Male)	7/109	2/66	5/43	0.124†
Smoking				0.974
Yes	92(79.3%)	54(58.7%)	38(41.3%)	
No	24(20.7%)	14(58.3%)	10(41.7%)	
Alcohol				0.196
Heavy	37(31.9%)	26(70.3%)	11(29.7%)	
Social	47(40.5%)	26(55.3%)	21(44.7%)	
No	32(27.6%)	16(50.0%)	16(50.0%)	
Betel quid				0.885
Yes	83(71.6%)	49(59.0%)	34(41.0%)	
No	33(28.4%)	19(57.6%)	14(42.4%)	
Primary tumor sites				0.550†
Oral cavity	104(89.7%)	62(59.6%)	42(40.4%)	
Others	12(10.3%)	6(50.0%)	6(50.0%)	
Histological features				0.261
Well differentiated	8(6.9%)	4(50.0%)	4(50.0%)	
Moderately differentiated	82(70.7%)	52(63.4%)	30(36.6%)	
Poorly or undifferentiated	26(22.4%)	12(46.2%)	14(53.8%)	
Perineural invasion				0.307
Yes	26(22.4%)	18(69.2%)	8(30.8%)	
No	90(77.6%)	50(55.6%)	40(44.4%)	
Angiolymphatic invasion				0.054
Yes	26(22.4%)	20(76.9%)	6(23.1%)	
No	90(77.6%)	48(53.3%)	42(46.7%)	
Extracapsular invasion				0.359†
Yes	11(9.5%)	8(72.7%)	3(27.3%)	
No	105(90.5%)	60(57.1%)	45(42.9%)	
Pathological stage				0.662
Stage I-II	54(46.6%)	30(55.6%)	24(44.4%)	
Stage III-IV	62(53.4%)	38(61.3%)	24(38.7%)	
Postoperative radiotherapy				0.870
Yes	53(45.7%)	32(60.4%)	21(39.6%)	
No	63(54.3%)	36(57.1%)	27(42.9%)	
Local recurrence				0.307
Yes	26(22.4%)	18(69.2%)	8(30.8%)	
No	90(77.6%)	50(55.6%)	40(44.4%)	
Survival status				0.338
Alive	101(87.1%)	57(56.4%)	44(43.6%)	
Death	15(12.9%)	11(73.3%)	4(26.7%)	

^†^Fisher’s exact test.

**Table 3:** Cox proportional hazard model.

Variables	No. of patients (N = 116)	Relative Risk	*P* value	95% Confidence Interval	
				Lower limit	Upper limit
Age					
<50 years*	49	1.000			
>=50 years	67	1.306	0.624	0.450	3.790
Gender					
Female*	7	1.000			
Male	109	0.412	0.407	0.050	3.357
Primary tumor site					
Oral cavity*	104	1.000			
Others	12	0.573	0.594	0.074	4.445
MSI					
No*	72	1.000			
Yes	44	2.242	0.168	0.711	7.075
LOH					
No*	48	1.000			
Yes	68	1.115	0.865	0.317	3.921
Pathological stage					
I-II*	54	1.000			
III-IV	62	4.496	0.022	1.237	16.34

^*^Reference group.

Abbreviation: MSI, microsatellite instability; LOH: loss of heterozygosity.

**Table 4:** Factors associated with local recurrence based on logistic regression model.

Variables	No. of margins (N = 351)	Odds Ratio	*P* value	95% Confidence Interval	
				Lower limit	Upper limit
Age					
<= 50 years	161	1.111	0.803	0.484	2.550
> 50 years*	190	1.000			
Gender					
Female	12	2.012	0.421	0.367	11.03
Male*	339	1.000			
Pathological stage					
Stage III, IV	191	2.520	0.044	1.026	6.186
Stage I, II*	160	1.000			
Primary tumor site			0.105		
Hypopharynx	13	4.002	0.061	0.940	17.04
Oropharynx	23	0.385	0.388	0.044	3.357
Oral cavity*	315	1.000			
MSI					
Yes	40	15.14	<0.001	6.451	35.53
No*	311	1.000			
LOH					
Yes	74	1.295	0.577	0.522	3.216
No*	277	1.000			

^*^Reference group.

Abbreviation: MSI: microsatellite instability; LOH: loss of heterozygosity.
