# Supplementary material for: Microsatellite alteration in head and neck squamous cell carcinoma patients from a betel quid-prevalent region
Source: Sci Rep. 2016 Mar 24;6:22614. doi: 10.1038/srep22614 (PMC4806345; doi:10.1038/srep22614)
Supplement: Supplementary Information [file srep22614-s1.doc]

Microsatellite alteration in head and neck squamous cell carcinoma patients from a betel quid-prevalent region

Jin-Ching Lin, Chen-Chi Wang, Rong-San Jiang, Wen-Yi Wang, Shih-An Liu

Supplementary table 1. Primers used for microsatellite alteration analysis

| Marker | Forward (5’🡪 3’) | Reverse (3’ 🡪 5’) | Size (bp) | Repeat | Location |
| --- | --- | --- | --- | --- | --- |
| D2S206 | TTAAAAATTAAGTAGGCTTTTGGTT | GTCCTCATGTGTTTATGCTGT | 238 | Dinucleotide | 2q33-37 |
| D3S1079 | GGGAGATAGGTAGTATCATCT | ATCTACCATTAAGGCAACCTG | 136 | Dinucleotide | 3p25 |
| D3S1234 | CCTGTGAGACAAAGCAAGAC | GACATTAGGCACAGGGCTAA | 111 | Dinucleotide | 3p21.1-3p14.2 |
| D3S1300 | ACAAAGGAACGTCATGTGGTAGG | GCTGTTTATTCTTCGTGGAATGCC | 155 | Dinucleotide | 3p21.1-3p14.2 |
| THRB | GATCACAAGGATGCTAGAGT | TCAAAGGAGTCAGGCTGTAG | 197 | Dinucleotide | 3p24.1-3p22 |
| D9S1748 | CACCTCAGAAGTCAGTGAGT | GTGCTTGAAATACACCTTTCC | 130 | Dinucleotide | 9p21-9p21 |
| IFNA.PCR2 | TGCGCGTTAAGTTAATTGGTT | GTAAGGTGGAAACCCCCACT | 138 -150 | Dinucleotide | 9p22-9p22 |
| D21S11 | ATATGTGAGTCAATTCCCCAAG | TGTATTAGTCAATGTTCTCCAG | 223 | Tetranucleotide | 21q21 |
| D21S1433 | GCGGGCACTGTAGTCTCAG | CTATTTTCAGGCCAAGCCTT | 240 | Tetranucleotide | 21p-q12 |
| D21S236 | CCCAAATAAAAAAGAGAACAG | CTAAAGAGGACTTCAGAGTAAGG | 104 | Dinucleotide | 21q11.1 |
